# Evolutionary action score identifies a subset of *TP53* mutated myelodysplastic syndrome with favorable prognosis

**DOI:** 10.1038/s41408-021-00446-y

**Published:** 2021-03-06

**Authors:** Rashmi Kanagal-Shamanna, Guillermo Montalban-Bravo, Panagiotis Katsonis, Koji Sasaki, Caleb A. Class, Elias Jabbour, David Sallman, Anthony Michael Hunter, Christopher Benton, Kelly S. Chien, Rajyalakshmi Luthra, Carlos E. Bueso-Ramos, Tapan Kadia, Michael Andreeff, Rami S. Komrokji, Najla H Al Ali, Nicholas Short, Naval Daver, Mark J. Routbort, Joseph D. Khoury, Keyur Patel, Irene Ganan-Gomez, Yue Wei, Gautam Borthakur, Farhad Ravandi, Kim-Anh Do, Kelly A. Soltysiak, Olivier Lichtarge, L. Jeffrey Medeiros, Hagop Kantarjian, Guillermo Garcia-Manero

**Affiliations:** 1 Department of Hematopathology and Molecular Diagnostics, Division of Pathology and Lab Medicine, The University of Texas MD Anderson Cancer Center, Houston, TX United States; 2 Department of Leukemia, The University of Texas MD Anderson Cancer Center, Houston, TX United States; 3 Department of Molecular and Human Genetics, Baylor College of Medicine, Houston, TX United States; 4 Department of Biostatistics, The University of Texas MD Anderson Cancer Center, Houston, TX United States; 5 Malignant Hematology Department, H. Lee Moffitt Cancer Center, Tampa, FL United States

**Keywords:** Translational research, Myelodysplastic syndrome

Dear Editor,

The prognosis of *TP53*-mutated myelodysplastic syndromes (MDS) can be heterogeneous. *TP53*-mutated MDS with low variant allele frequency (VAF), without complex karyotype (CK), and those with mono-allelic *TP53* alterations have significantly improved outcomes^1–3^. *TP53* mutations are diverse and distributed across the codons of the entire coding region^4^. Different types of *TP53* mutations lead to distinct functional consequences (such as oncogenic gain-of-function, protein loss-of-function with dominant-negative effect etc^5–7^), that likely influence disease biology and outcome, either independently or by influencing known variables such as VAF and allelic state^2,3^. Until now, the relationship between various *TP53* mutations and genomic/phenotypic features including outcomes is not well-characterized. This knowledge is important to assess the efficacy of novel therapeutic strategies that restore TP53 function^8^.

Evolutionary Action score (EAp53) is a computationally-derived score to quantify the deleterious impact of different missense *TP53* mutations based on (A) phylogenetic divergence of the mutated sequence position [evolutionary trace (ET)] and (B) perturbation due to amino acid (AA) substitution^9^. EAp53 score ranges between 0 and 100, a higher score indicates a worse impact, and 0 indicates wild-type function. EAp53 score has been shown to be an objective, reliable prognostic biomarker in patients with head and neck (H&N) and colorectal cancers^10–13^. Here, we used the EAp53 scoring system to evaluate the impact of different types of missense *TP53* mutations on clinico-pathologic and genomic features in MDS.

We identified 270 patients with newly-diagnosed MDS or oligoblastic AML (<30% blasts) with ≥1 missense *TP53* mutation(s) at baseline detected by next-generation sequencing (Fig. 1A). The median *TP53* VAF was 33.9 (1–94.4); 165 (61%) had multi-allelic *TP53* alterations. Majority were treated with hypomethylating agents (HMA). Informed consent was obtained, the study was performed per institutional-approved protocols in accordance with the Declaration of Helsinki. See Supplementary Materials for detailed methodology.

**Fig. 1:**
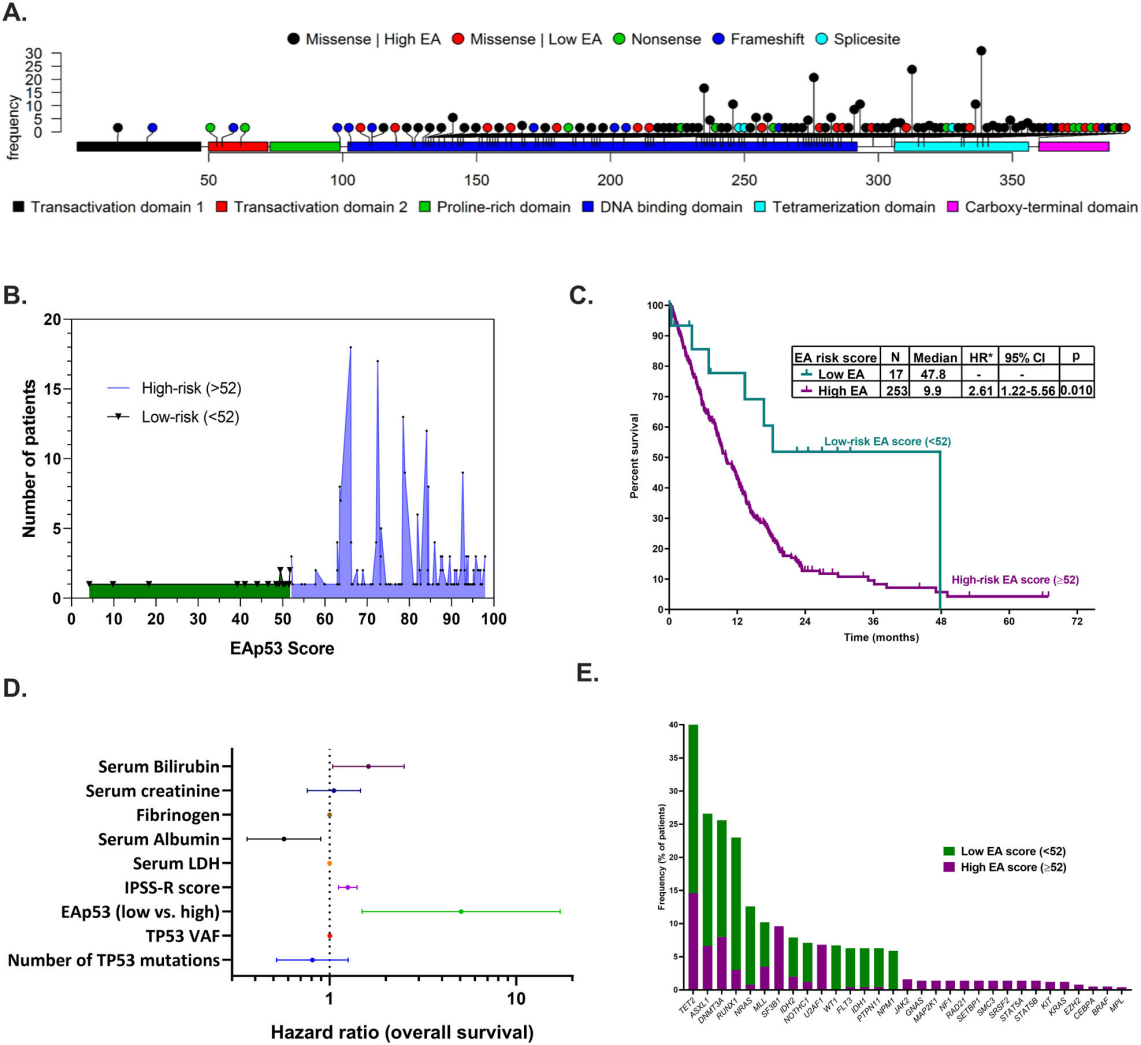
Correlations between EAp53 score and TP53 mutational characteristics, clinicopathologic features and outcome. **A** Lollipop plot showing the frequency distribution of missense *TP53* mutations and associated concurrent non-missense mutations. **B** Spectrum of EAp53 scores of the TP53 mutations noted within our MDS cohort: the majority had a high (>52) EAp53 score. **C** Using RPART, an EAp53 score of 52 provided an optimal cut-off based on overall survival in MDS patients. **D** The multivariate model identified EAp53 score, R-IPSS risk score, and serum bilirubin to be an independent predictor for worse overall survival. **E** Mutational frequencies of genes in the cohort separated by EAp53 risk category. Low-risk EAp53 MDS patients had a significantly higher frequency of mutations in *NRAS* and *RUNX1*, and a trend for higher frequencies in *NPM1*, *WT1*, and *ASXL1* mutations.

Baseline characteristics are in Table S1. The median EAp53 score was 79 (4.2–97.9) (Fig. 1B). A higher EAp53 score correlated with worse OS (*p* = 0.087; HR 1.06 per 10-point increase [95%CI:1.01–1.13]). Using Recursive-Partitioning-And-Regression-Trees, EAp53 score >52 predicted for worse OS (Fig. 1C) generating 2 risk-groups: low-EAp53 [EAp53 ≤ 52; *n* = 17 (6%)] and high-EAp53 [>52; *n* = 253, 94%]. The median OS for low-EA-MDS vs. high-EA-MDS was 47.8 vs. 10 months (*p* = 0.01; HR: 2.6 [1.22–5.56]). EAp53 score of 75, previously described in *TP53*-mutated H&N squamous cell carcinoma, did not show a survival difference in MDS. By univariate analysis, high-EAp53 (>52), *TP53* VAF, number of *TP53* mutations, IPSS-R score, CK/monosomal karyotype (MK), higher serum LDH and creatinine, lower platelet, hemoglobin, and serum albumin associated with worse OS. Neither *TP53* allele state nor del(17p) associated with OS. By multivariable analysis, the EAp53 risk retained the independent predictive value for OS along with IPSS-R score and serum albumin, but not *TP53* VAF or the number of *TP53* mutations (CK excluded due to a strong association with EAp53 score; Fig. 1D**;** Table S2). EAp53 risk was the only independent predictor of AML transformation. EAp53 risk did not affect transformation-free survival, relapse-free survival, overall response, and complete remission rates (Table S3).

Next, we explored the clinico-pathologic and cytogenomic differences between low-EA-MDS and high-EA-MDS (Table S4). Higher EAp53 score (as a continuous variable) positively correlated with multiple *TP53* mutations (*p* = 0.00062), higher platelet (*p* = 0.041), and serum fibrinogen (*p* = 0.009), and negatively correlated with concurrent *RUNX1* (*p* = 0.038) and *EZH2* (*p* < 0.001) mutations. When stratified, low-EA-MDS had fewer cytogenetic abnormalities (median 3 vs. 7, *p* = 0.019), lower frequency of CK (*p* = 0.0241), and MK (*p* = 0.0043). High-EAp53-MDS had a higher frequency of multiple *TP53* mutations (32% vs. 6%, *p* = 0.027) and multi-allelic *TP53* alterations (63% vs. 29%, *p* = 0.0087), suggesting that the type of mutation dictates the degree of karyotypic complexity. Patients with gain-of-function *TP53* mutations (R175, R248, R273, all noted only in high-EA-MDS) showed no significant outcome difference compared to rest (Fig. S1). Across all genes, the median mutation number (including *TP53*) in low-EAp53 and high-EAp53 was 3 and 1 (*p* = 0.000002). A higher proportion of low-EAp53 patients had additional gene mutations (63% vs. 33%; *p* = 0.05), involving *NRAS* and *RUNX1* (*p* = 0.02) and a trend for higher frequencies of *NPM1*, *WT1*, and *ASXL1* mutations (Fig. 1E**;** Fig. S2). There were no significant differences in the median *TP53* VAF, distribution of IPSS-R, therapy-related, or treatment characteristics.

The observed distinctive clinical, cytogenetic, and mutation characteristics provide support to the clinical validity of EAp53 scoring and confirm that low and high-EAp53 do not reflect different positions on the early to late disease trajectory. The presence of at least 1 additional gene mutation, frequently *NRAS*, in low-EA-MDS corroborates the leukemogenic role of RAS. These additional hits potentially modify the phenotype and outcome of low-EA-MDS. The need for additional hits in high-EA-MDS is abrogated by chromosomal aneuploidies, involving chromosomes 17 and 5, that harbor negative regulators of the RAS pathway^14^.

We then assessed the downstream effect of the EAp53 score using immunohistochemical TP53 protein expression (low-EAp53: *n* = 10; median EAp53: 27.9; high-EAp53: *n* = 20; 84.8). The median H-scores (multiplied score of percent positivity and intensity) for wild-type (6.4), low-EAp53 (47.5), and high-EAp53 (157.5) were significantly different (*p* < 0.05) (Fig. 2A–D). These results are in accord with the mRNA studies in squamous cell carcinoma cells where low-EAp53 cells partly retained residual TP53 function^10,11^. H-score correlated with *TP53* VAF (*p* = 0.00015; rho(ρ) = 0.61).

**Fig. 2:**
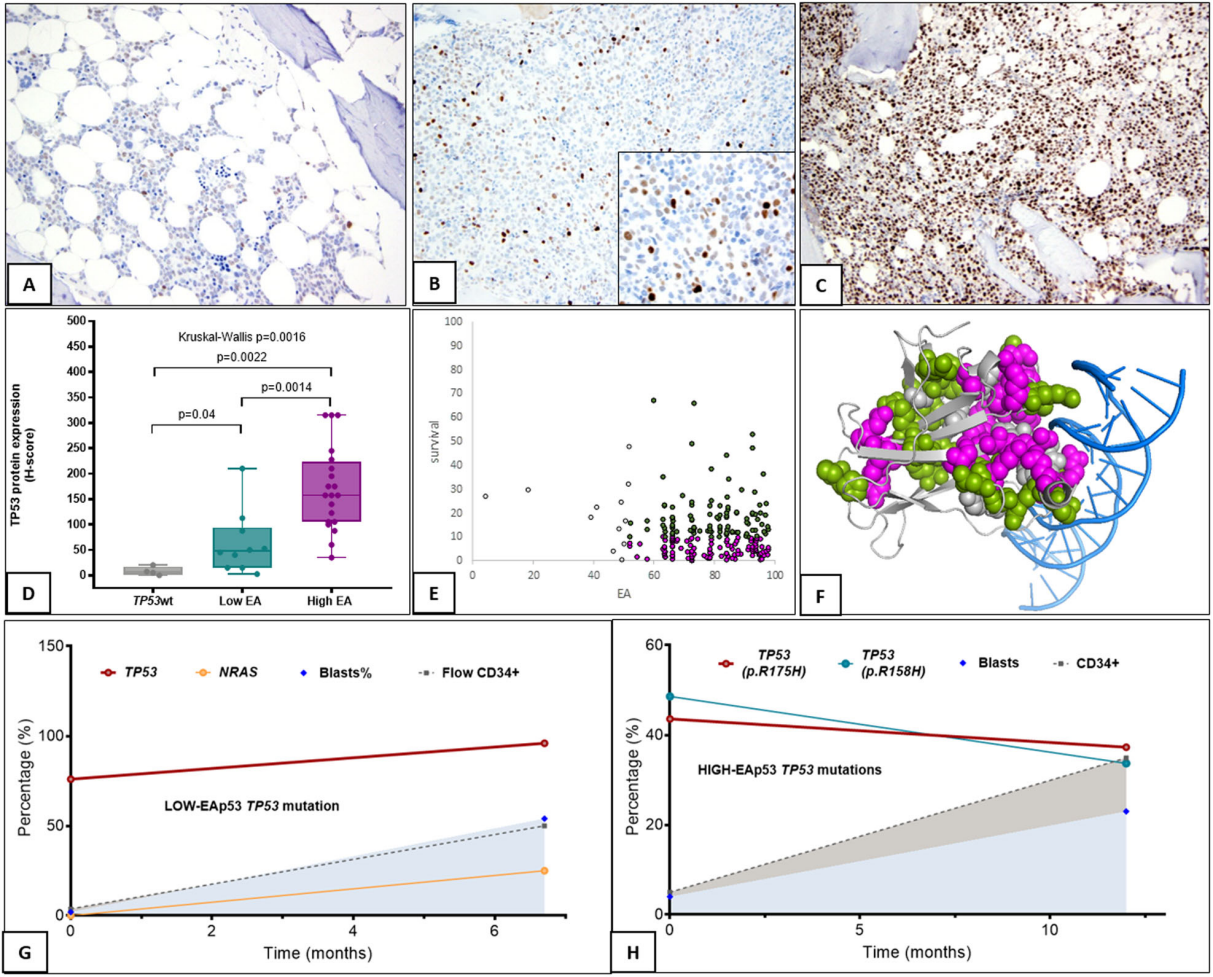
EAp53 score and TP53 protein expression (A-D), TP53 protein structural analysis (E-F) and sequential NGS comparing mutational dysnamics in low-EA-MDS and high-EA-MDS. **A** TP53 protein expression by immunohistochemistry: immunohistochemical staining pattern for in low-EA-MDS with diploid karyotype shows weak staining in ~80% of cells (**B**) low-EA-MDS with complex karyotype shows the dual population of cells: strong positive cells in ~5% and weak positive cells in 10% of all cells; inset shows staining at ×1000 magnification (**C**) IHC staining pattern in high-EA-MDS shows strong positivity in >50% of cells (high H-score). **D** Median H-scores of TP53 protein expression by IHC showed significant differences between *TP53* wild-type MDS, low-EA-MDS, and high-EA-MDS. **E** Graph demonstrating the variability of overall survival of patients with the same EAp53 scores attributed to the different structural location of these mutants on the protein: survival time is plotted against the Evolutionary Action score, for 215 patients who were divided into 113 patients with poor survival (pink dots) and 102 patients with good survival (green dots) using a threshold of 10 months. **F** A cartoon representation of the TP53 core domain structure bound to DNA (PDB ID of 4HJE, visualized by PyMOL) with residues mutated mostly in patients with poor survival represented by pink atomic spheres, residues mutated mostly in patients with good survival represented by green atomic spheres, and residues with equal numbers of patients with poor and good survival represented by white atomic spheres. Sequential NGS analysis of **G**. low-EA-MDS showing persistence of *TP53* mutation with additional concurrent *NRAS* mutation at AML transformation and (**H**) high-EA-MDS showing persistence of *TP53* mutations without new mutations at AML transformation. The gray color is the area under the track of CD34+ cells over time detected by flow cytometry. The blue color is the area under the blast percentage tracked over time by morphologic evaluation.

Since protein function is further modulated by the 3D location of the residue, we performed protein structural analysis using the crystal structure of the TP53-core-domain in complex with DNA (PDB ID: 4HJE; PyMOL molecular visualization). We hypothesized that this may explain the variable survival rates noted in some high-EA-MDS patients with similar EAp53 scores. All the *TP53* mutations of this cohort mapped to the evolutionarily important sites of the TP53-core-domain. When segregated based on survival of 10 months, *TP53* variants with poor-survival (OS < 10 months) formed two clusters: a large cluster interfacing the DNA-binding site and a small cluster formed by residues V157, Y220, L257, and E258, showing that structure location further stratifies the outcome (Fig. 2E, F). Analysis with different survival cut-offs yielded the same results.

Following this, using serial NGS, we compared the mutational dynamics of low vs. high-EA *TP53* mutations during disease evolution and therapy. Among 9 low-EAp53, 2 of 3 (67%) who achieved at least partial response showed mutation clearance. The remaining showed persistence of the same *TP53* mutation with additional mutations in *NRAS* (Fig. 2G)*, KRAS, RUNX1, IDH1*, and *JAK2*. None acquired new *TP53* mutations. Among 36 high-EAp53 MDS, 5 of 11 (45%) who achieved at least morphologic CR showed mutation clearance. Rest had persistence of the original *TP53* mutation(s) (Fig. 2H); 1 acquired 3 additional *TP53* mutations (also high-EA). Only 2 patients (8%) acquired new mutations in *NRAS*, *IDH1*, and *TET2*.

Finally, we verified the biological relevance of EAp53 scoring using other independent computational methods. CADD and REVEL segregated the same prognostic subgroups (but not DANN, Polyphen 2, MutPred, PROVEAN, SIFT; Fig. S3). To validate the EAp53 cut-off of 52, we used an independent single-center cohort of 62 MDS patients, selected using the same criteria and treated using HMAs. There were 3 (5%) low-EA-MDS patients [p.Y220H, p.F134L, p.R209W] with a longer median OS (112 vs. 32 months, *p* = 0.25) compared to high-EA-MDS (Fig. S4). CADD and REVEL could not separate these patients, suggesting that the EAp53 method was superior. When study and validation cohorts were merged, all 3 methods were concordant [EAp53, *p* = 0.0103; REVEL, *p* = 0.03; CADD *p* = 0.006; Fig. S5].

The study has a few limitations. Although this is a large retrospective study, the inherent low frequency of low-EAp53 MDS (~6%) warrants validation in multi-center cohorts. While the possibility that some of the low-EAp53 variants represent rare single nucleotide polymorphisms (SNP) cannot be completely excluded, to the best of our knowledge, all low-EAp53 variants were clinically reported by the laboratory after extensive curation using literature, online databases including COSMIC, dbSNP, 1000 genome, EXAC, ClinVar and in-silico prediction tools. Repeat NGS on 9 (53%) patients showed clearance or significant variations in the *TP53* VAFs, strongly suggesting somatic origin. *TP53* VAF was not independently prognostic in this study. Unlike other reports^1,2^, we note that this cohort is unique because it excluded patients with nonsense/frameshift *TP53* mutations that are likely to have higher VAF and multi-allelic *TP53* alterations due to a loss-of-function phenotype. Further, VAFs were not normalized based on copy number. The study did not assess copy-neutral loss-of-heterozygosity that could explain the lack of association with *TP53* allele status.

In conclusion, this is the first study to show the independent prognostic value of the EAp53 score, thereby expanding the previously established genomic attributes impacting the outcomes of *TP53*-mutated MDS^1–3^. While VAF and karyotype are dependent on the aspirate quality (often compromised by fibrosis in *TP53*-mutated MDS) and vary with disease evolution and therapy, EAp53 score is mutation-dependent, stable predictive biomarker, not influenced by therapy or time for baseline risk-stratification. These findings are important in lieu of novel mutation type-specific therapeutic strategies^7,15^. Low-EAp53 mutants may benefit from strategies that utilize residual TP53 function while small molecules, such as APR-246 and COTI-2, which restore TP53 function may be appropriate for high-EAp53 mutants^8^. Together with structural mapping, the EAp53 score can guide treatment. Overall, the study shows that the EAp53 score can identify prognostic subsets within *TP53*-mutated MDS and facilitate a personalized therapeutic approach.

## Supplementary information

Supplemental Material

Supplemental Figure S1.

Supplemental Figure S2.

Supplemental Figure S3.

Supplemental Figure S4.

Supplemental Figure S5.

## Data Availability

The datasets generated during and/or analyzed during this study are not publicly available due to patient privacy concerns but are available from the corresponding author on reasonable request.
