# Supplementary material for: Evolutionary action score identifies a subset of TP53 mutated myelodysplastic syndrome with favorable prognosis
Source: Blood Cancer J. 2021 Mar 6;11(3):52. doi: 10.1038/s41408-021-00446-y (PMC7936977; doi:10.1038/s41408-021-00446-y)
Supplement: Supplementary file 1 — Supplemental Material [file 41408_2021_446_MOESM1_ESM.docx]

**Evolutionary Action Score Identifies a Subset of *TP53*-Mutated Myelodysplastic Syndrome with Favorable Prognosis**

Rashmi Kanagal-Shamanna^1*^, Guillermo Montalban-Bravo^2^, Panagiotis Katsonis^3^, Koji Sasaki^2^, Caleb A. Class^4^, Elias Jabbour^2^, David Sallman^5^, Anthony Michael Hunter^5^, Christopher Benton^2^, Kelly S. Chien^2^, Rajyalakshmi Luthra^1^, Carlos E. Bueso-Ramos^1^, Tapan Kadia^2^, Michael Andreeff^2^, Rami S. Komrokji^5^, Najla Al Ali^5^, Nicholas Short^2^, Naval Daver^2^, Mark J. Routbort^1^, Joseph D. Khoury^1^, Keyur Patel^1^, Irene Ganan-Gomez^2^, Yue Wei^2^, Gautam Borthakur^2^, Farhad Ravandi^2^, Kim-Anh Do^2^, Kelly A. Soltysiak^2^, Olivier Lichtarge^3^, L. Jeffrey Medeiros^1^, Hagop Kantarjian^2^, Guillermo Garcia-Manero^2^

^1^Department of Hematopathology, The University of Texas MD Anderson Cancer Center, Houston, Texas

^2^Department of Leukemia, The University of Texas MD Anderson Cancer Center, Houston, Texas ^3^Department of Molecular and Human Genetics, Baylor College of Medicine, Houston, Texas

^4^Department of Biostatistics, The University of Texas MD Anderson Cancer Center, Houston, Texas

^5^Malignant Hematology Department, H. Lee Moffitt Cancer Center, Tampa, Florida, United States.

**Running Title:** Evolutionary Action Score of *TP53* Mutations

^*^**Corresponding author:**

Rashmi Kanagal-Shamanna, MD

The University of Texas MD Anderson Cancer Center

Department of Hematopathology

1515 Holcombe Boulevard

Houston, Texas 77030

United States of America

Phone: 713-745-4947

E-mail: [RKanagal@mdanderson.org](mailto:RKanagal@mdanderson.org)

**METHODS**

**Patient Selection**

All patients presenting to our institution (between 2012-2018) with newly diagnosed MDS or AML with <30% blasts [MDS-EB-T, per NCCN guidelines ^1^], with at least 1 missense *TP53* mutation were included. The latter (oligoblastic AML, 20-29%) were included for the following reasons. First, the NCCN recognizes “MDS-EB-T” to be within the spectrum of MDS based on similarity in clinical course ^1,2^. The currently validated prognostic models for MDS, including IPSS-R, incorporate this patient subgroup ^3^. Second, the current understanding of genomic data suggests that *TP53*-mutated MDS/oligoblastic AML represent a spectrum of the same disease, and a cut-off of 20% seems arbitrary ^4^. Hence, we expanded the blast cut-off to 30% for this study, similar to the International Working Group for Prognosis in MDS (IWG-PM). All patients underwent bone marrow (BM) morphologic evaluation using World Health Organization criteria ^4^. Conventional karyotyping was performed using standard techniques ^5^. The prognostic risk was calculated using R-IPSS ^3,6^. Patients that developed *TP53* mutations over the disease course were excluded. Informed consent was obtained, and all studies were performed based on the institutional approved protocols in accordance with the Declaration of Helsinki.

**Molecular Analysis**

All patients underwent next-generation sequencing (NGS)-based *TP53* mutation analysis using 53/28/81-gene panels within a Clinical Laboratory Improvement Amendments (CLIA)-certified Laboratory ^5,7,8^. Majority included the entire *TP53* coding region: the entire *TP53* coding region (exons 2-11, codons 1-394) was sequenced in 138 (51%) and exons 2-11 (codons 1-25, 80-394) in 83 (31%). In ~18% (49 patients), only hotspot regions were sequenced [exons 2 (1-12), 4 (69-112), 5-7 (126-253), 8 (267-306), 10 (332-342)]. The analytical sensitivity was 1% with adequate coverage. Since matched germline samples were not sequenced, the somatic nature was inferred based on the data in the literature, institutional and online databases such as Exome Aggregation Consortium [ExAC], dbSNP 137/138, and 1000 Genomes Project and VAFs ^8^. *FLT3*-ITD mutations were evaluated by PCR-based capillary electrophoresis.

**EAp53 Scoring**

EAp53 scores for all missense *TP53* mutations were obtained from the EAp53 server at <http://mammoth.bcm.tmc.edu/EAp53>. Higher scores represent alterations that are more deleterious. For patients with >1 *TP53* missense mutations, the highest EAp53 score was considered irrespective of the VAF. The optimal cut-off EAp53 score for distinguishing a low versus high EAp53 score for *TP53* mutations in MDS was obtained as described in Results.

**Immunohistochemical (IHC) Staining for TP53 Protein Expression**

IHC was performed using monoclonal anti-TP53 antibody clone DO-7 (Dako, Carpinteria, CA, USA) on BM biopsy sections using standard techniques ^9^. IHC staining was scored independently by two hematopathologists (RK-S, CB-R) in a blinded fashion. In addition to percent cells showing nuclear positivity of all nucleated cells (at least 5,000 nucleated cells were counted), cases were semi-quantitatively scored for intensity (negative=0/1; weak=2; moderate=3; strong=4). An H-score was calculated by multiplying percent positivity and the intensity scores. In cases showing dual populations of cells with different intensities, an average H-score from both populations was calculated.

**Statistical Analysis**

Responses were evaluated using the 2006 IWG-PM criteria ^10^. OS was defined as time from diagnosis to death/last follow-up. The time-to-transformation was calculated for WHO-defined MDS patients from diagnosis to AML. Relapse-free survival (RFS) was defined as time from response until relapse/death. Transformation-free survival (TFS) was defined as time from diagnosis to AML transformation/death. Patients alive at their last follow-up were censored. The median OS, RFS and time-to-transformation were evaluated by the Kaplan-Meier method. Univariate Cox proportional hazards analyses were used to identify associations between risk factors and survival followed by multivariate Cox analyses. In addition to clinico-pathologic parameters, *TP53* VAF and EAp53 scores were correlated to clinical outcomes. The optimal EAp53 cutoff was determined using recursive partitioning and regression trees (RPART) based on the CART algorithm ^11-13^. RPART identified the cutoff(s) that maximized the homogeneity of survival times within groups (or minimized the within-node deviancy). A complexity parameter of 0.01 was used. Logistic regression (for continuous) and Fisher’s exact test (for categorical variables) were used to study the association of overall response (OR) or complete response rates (CR) and risk factors. Statistical analysis was performed using R version 3.5.1 ^14^.

**TP53 Protein 3D Structure Analysis**

TP53 protein structural analysis was conducted using the PyMOL molecular visualization system and the crystal structure of the TP53 core domain in complex with DNA (PDB ID of 4HJE) ^15^. The importance of the protein residues estimated by the Evolutionary Trace approach ^16^ was represented using a color scale, red (most important) to green (least important), using the PyETV plugin ^17^. To correlate survival with molecular structural location, we performed structural analysis on 215 high-risk EAp53 (>52) patients who were divided into 2 groups based on survival cut-off of 10 months: 113 patients with <10 months and 102 with ≥10-month survival. Each TP53 protein residue was considered as high- or low-risk when >50% of the patients had poor or good survival, respectively. Residues with equal numbers of patients with poor and good survival were considered “neutral”. Using more extreme cut-offs (<5 months and >15 months, <2.5 months and >20 months, <1 month and >30 months) yielded the same results.

**References**

1. Greenberg, P.L. *et al.* Myelodysplastic Syndromes, Version 2.2017, NCCN Clinical Practice Guidelines in Oncology. *J Natl Compr Canc Netw* **15**, 60-87 (2017).

2. DiNardo, C.D. *et al.* Interactions and relevance of blast percentage and treatment strategy among younger and older patients with acute myeloid leukemia (AML) and myelodysplastic syndrome (MDS). *Am J Hematol* **91**, 227-232 (2016).

3. Greenberg, P.L. *et al.* Revised international prognostic scoring system for myelodysplastic syndromes. *Blood* **120**, 2454-65 (2012).

4. Arber, D.A. *et al.* The 2016 revision to the World Health Organization classification of myeloid neoplasms and acute leukemia. *Blood* **127**, 2391-405 (2016).

5. Kanagal-Shamanna, R. *et al.* Myeloid neoplasms with isolated isochromosome 17q demonstrate a high frequency of mutations in SETBP1, SRSF2, ASXL1 and NRAS. *Oncotarget* **7**, 14251 (2016).

6. Greenberg, P. *et al.* International scoring system for evaluating prognosis in myelodysplastic syndromes. *Blood* **89**, 2079-88 (1997).

7. Montalban-Bravo, G. *et al.* NPM1 mutations define a specific subgroup of MDS and MDS/MPN patients with favorable outcomes with intensive chemotherapy. *Blood Adv* **3**, 922-933 (2019).

8. Kanagal-Shamanna, R. *et al.* Principles of analytical validation of next-generation sequencing based mutational analysis for hematologic neoplasms in a CLIA-certified laboratory. *Expert Rev Mol Diagn* **16**, 461-72 (2016).

9. Assi, R. *et al.* P53 protein overexpression in de novo acute myeloid leukemia patients with normal diploid karyotype correlates with FLT3 internal tandem duplication and worse relapse-free survival. *Am J Hematol* **93**, 1376-1383 (2018).

10. Cheson, B.D. *et al.* Clinical application and proposal for modification of the International Working Group (IWG) response criteria in myelodysplasia. *Blood* **108**, 419-25 (2006).

11. Therneau, T., Atkinson, B. & Ripley, B. Rpart: Recursive Partitioning and Regression Trees, R package version 4.1-13. 2018. (2019).

12. Breiman, L.F., Friedman, J., Olshen, S. & Stone, C. CJ, 1984. Classification and regression trees. *Pacific Grove, Kalifornien* (1983).

13. Akaike, H. A new look at the statistical model identification. in *Selected Papers of Hirotugu Akaike* 215-222 (Springer, 1974).

14. Team, R.C. R: A language and environment for statistical computing. R Foundation for Statistical Computing, Vienna, Austria. 2012. [*http://www.R-project.org/*](http://www.R-project.org/) (2018).

15. Chen, Y. *et al.* Structure of p53 binding to the BAX response element reveals DNA unwinding and compression to accommodate base-pair insertion. *Nucleic Acids Res* **41**, 8368-76 (2013).

16. Mihalek, I., Reš, I. & Lichtarge, O. A family of evolution–entropy hybrid methods for ranking protein residues by importance. *J Mol Biol* **336**, 1265-1282 (2004).

17. Lua, R.C. & Lichtarge, O. PyETV: a PyMOL evolutionary trace viewer to analyze functional site predictions in protein complexes. *Bioinformatics* **26**, 2981-2982 (2010).

**Supplemental Figure Legends**

**Supplemental Figure S1**. MDS patients with gain-of-function *TP53* mutations (R175, R248, R273, all noted only in high-EA-MDS) showed no significant outcome difference compared to rest (median OS, 10.2 vs. 11.1 months; p=0.77).

**Supplemental Figure S2.** Somatic mutation spectra of low and high EAp53 AML/MDS patients were different; high EAp53 patients lacked additional mutations in other genes (median of 1 mutation per case) while low EAp53 patients had a median of 3 additional mutations. High EAp53 patients had more frequent chromosomal aberrations.

**Supplemental Figure S3.** Independent comparison of EA scoring system, CADD and REVEL algorithms to score the mutation effect to identify a prognostic cut-off value. All 3 algorithms were able to segregate the same set of patients based on survival analysis with a significant p-value.

**Supplemental Figure S4.** Summary of the results of the other algorithms (DANN, MutPred, MPC, Eigen raw, Polyphen 2, PROVEAN, Mutation Assessor and SIFT algorithms that were not consistently able to separate the patients based on survival.

**Supplemental Figure S5.** When the EAp53 cut-off score of 52 was used in an independent single-center MDS cohort of 62 MDS patients, selected using the same selection and exclusion criteria, 3 (5%) of patients were classified as low-EAp53. Patients with a low EA score with a cut-off of 52 had a longer OS (112 months vs. 32 months) but p-value was not significant due to too few numbers.

**Supplemental Table Legends**

**Supplemental Table S1.** Patient characteristics of *TP53* mutated MDS patients

**Supplemental Table S2.** Multivariable model incorporating clinical and mutational characteristics of *TP53* mutation

**Supplemental Table S3.** Comparison of survival outcomes of *TP53* mutated MDS patients with low and high risk EAp53 scores

**Supplemental Table S4.** Comparison of clinicopathologic characteristics between *TP53* mutated AML/MDS patients with low EA and high EAp53 scores. P-values were calculated using the two-sample t-test (continuous variables), Fisher’s exact test (2 categories), or chi-squared test (more than 2 categories).

**Supplemental Table S1. Patient characteristics of *TP53* mutated MDS patients**

| **Characteristics** | **MDS** (n=270) |
| --- | --- |
| Age (median/ range) | 68.3 (18-90) |
| Gender: Female/ male | 102 (37%)/ 168 (62%) |
| Hemoglobin (g/dL) | 9.0 (6.4-16.2) |
| Platelet **(x10^9^/L)** | 49 (2-4816) |
| Absolute Neutrophil Count **(x10^9^/L)** | 1.1 (0-19.8) |
| PB blast% (median/ range) | 1 (0-25) |
| BM Blast% (median/ range) | 7 (0 to 28) |
| **IPSS-R categories** |  |
| Very-low/Low/Intermediate/High/Very-high | 9 (3.3%)/ 19 (7%)/ 19 (7%)/ 61 (22.6%)/ 162 (60%) |
| **WHO classification** |  |
| Therapy-related myeloid neoplasm | 122 (46%) |
| MDS-SLD-RS/ MDS-MLD-RS | 2 (0.7%)/ 7 (2.6%) |
| MDS-SLD / MDS-MLD | 1 (0.4%)/ 22 (8%) |
| MDS with excess blasts (MDS-EB) | 66 (24%) |
| MDS-Unclassifiable | 3 (1.1%) |
| MDS with isolated del5q | 2 (0.7%) |
| AML-MRC (MDS-EB-T) | 45 (16.7%) |
| **Karyotype** |  |
| Diploid | 23 (8.5%) |
| Complex | 219 (81.1%) |
| del(5q)/ monosomy 5 | 118 (43.7%) |
| del(7q)/ monosomy 7 | 94 (34.8%) |
| del(17p) | 93 (34.4%) |
| Monosomal karyotype | 232 (86%) |
| **Number of *TP53* mutations** |  |
| 1/ 2/ 3/ 4 | 189 (70%)/ 75 (28%)/ 5 (1.9%)/ 1 (0.4%) |
| ***TP53* allelic state: Monoallelic/ multiallelic** | 105 (39%)/ 165 (61%) |
| **Median VAF for *TP53* mutation** | 33.9 (1-94.4) |
| **EAp53 score** | 78.5 (4.2 – 97.94)) |
| High EA/ Low EA | 253 (94%)/ 17 (6%) |
| **Treatment regimens** | n=219 |
| Chemotherapy-based | 18 (8%) |
| Hypomethylating Agent (HMA)-Based | 167 (76%) |
| HMA + Chemo | 8 (4%) |
| HMA alone | 111 (51%) |
| HMA + other | 48 (22%) |
| Others | 3 (1%) |
| None | 22 (10%) |
| Number died | 192 (71%) |
| Transplant | 36 (13.3%) |

MDS, Myelodysplastic Syndrome; VAF, Variant Allele Frequency; R-IPSS, revised International Prognostic Scoring System; HMA, hypomethylating agent

**Supplemental Table S2.** **Multivariable model incorporating clinical and mutational characteristics of *TP53* mutation**

**OS**

| **Parameters** | **HR** | **lower .95** | **upper .95** | **p-value** |
| --- | --- | --- | --- | --- |
| Number of TP53 mutations | 0.8091 | 0.5207 | 1.2571 | 0.34604 |
| TP53 VAF | 1.0028 | 0.9936 | 1.012 | 0.55441 |
| **EAp53 (low vs. high)** | 5.0643 | 1.4928 | 17.1801 | **0.00925** |
| **IPSS-R score** | 1.2524 | 1.1184 | 1.4025 | **9.74E-05** |
| Serum LDH | 1 | 0.9996 | 1.0004 | 0.87182 |
| **Serum Albumin** | 0.57 | 0.362 | 0.8978 | **0.0153** |
| Fibrinogen | 1.0015 | 0.9999 | 1.003 | 0.06037 |
| Serum creatinine | 1.0547 | 0.7602 | 1.4634 | 0.74993 |
| **Serum Bilirubin** | 1.6136 | 1.0381 | 2.5082 | **0.03351** |
| **AML transformation** |  |  |  |  |
| **Parameters** | **HR** | **lower .95** | **upper .95** | **p-value** |
| TP53 VAF | 0.999 | 0.9871 | 1.011 | 0.8668 |
| **EAp53 (low vs. high)** | 7.4143 | 0.931 | 59.049 | **0.0584** |
| IPSS-R score | 1.1099 | 0.8702 | 1.416 | 0.401 |
| Complex cytogenetics | 3.0833 | 0.6095 | 15.596 | 0.1734 |
| Hemoglobin | 0.9122 | 0.7397 | 1.125 | 0.3906 |
| PB Platelet count | 0.9973 | 0.9921 | 1.003 | 0.3244 |

MDS, Myelodysplastic Syndrome; AML, Acute Myeloid Leukemia; VAF, Variant Allele Frequency; HR, Hazard Ratio

**Supplemental Table S3.** **Comparison of survival outcomes of *TP53* mutated MDS patients with low and high risk EAp53 scores**

|  | **Low EAp53**  Median (95% CI) | **High EAp53**  Median (95% Cl) | **p-value** |
| --- | --- | --- | --- |
| Overall Survival* | 47.8 (13.3-NA) | 10.0 (8.8-12.2) | **0.013** |
| Relapse-free Survival* | 12.6 (4.0-NA) | 6.6 (5.3-8.4) | 0.20 |
| Transformation-free survival* | 93.8 (NA-NA) | 49.5 (44.8-60.6) | **0.05** |
| Overall response rate** | 7/9 (78%) | 98/171 (57%) | 0.31 |
| Complete response rate** | 2/9 (22%) | 51/171 (30%) | 1 |

*p-value calculated by log-rank

**p-value calculated by Fisher’s exact test

CI, confidence interval; MDS, Myelodysplastic Syndrome

**Supplemental Table S4. Comparison of clinicopathologic characteristics between *TP53* mutated AML/MDS patients with low EA and high EAp53 scores. P-values were calculated using the two-sample t test (continuous variables), Fisher’s exact test (2 categories), or chi-squared test (more than 2 categories).**

| **Characteristics** | **Low EAp53 MDS** | **High EAp53 MDS** | **p-value** |
| --- | --- | --- | --- |
|  | n=17 | n=253 |  |
| **Age** | 72 (39-81) | 68 (18-90) | 0.92 |
| **Gender (M)** | 10 (59%) | 158 (62%) | 0.80 |
| **IPSS-R** |  |  | 0.28 |
| Very-low | 0 (0%) | 9 (4%) | ns |
| Low | 2 (12%) | 17 (7%) | ns |
| Intermediate | 3 (18%) | 16 (6%) | ns |
| High | 2 (12%) | 59 (23%) | ns |
| Very-high | 10 (59%) | 152 (60%) | ns |
| **Therapy-related** | 7 (41%) | 115 (45%) | 0.81 |
| **Karyotype** |  |  |  |
| Diploid | 2 (12%) | 23 (9%) | 0.6628 |
| Complex | 10 (59%) | 209 (83%) | 0.0241 |
| Non-complex | 7 (41%) | 44 (17%) | 0.0241 |
| del(5q)/ monosomy 5 | 6 (35%) | 148 (59%) | 0.4484 |
| del(7q)/ monosomy 7 | 5 (29%) | 116 (46%) | 0.4581 |
| Del(5q)/-5 & del(7q)/-7 | 4 (24%) | 84 (33%) | 1 |
| del(17p) | 4 (24%) | 89 (35%) | 0.4333 |
| Monosomal karyotype | 10 (59%) | 222 (88%) | 0.0043 |
| Median number of cytogenetic abnormalities | 3 (0-14) | 7 (0-27) | **0.019** |
| **Number of *TP53* mutations** |  |  |  |
| 1 | 16 (94%) | 173 (68%) | **0.027** |
| 2 or more | 1 (6%) | 80 (32%) |  |
| ***TP53* allelic state** |  |  | **0.0087** |
| Monoallelic | 12 (71%) | 93 (37%) |  |
| Multi-allelic | 5 (29%) | 160 (63%) |  |
| **Mutation location** |  |  |  |
| DNA-binding Domain | 16 (94%) | 249 (98%) | 0.46 |
| Oligomerization domain | 1 (6%) | 2 (0.8%) |  |
| Carboxy-terminal regulatory domain | 0 | 1 (0.4%) |  |
| Transactivation Domain | 0 | 1 (0.4%) |  |
| **Median VAF for TP53 mutation** | 23 (1-94) | 34 (1-94) | 0.68 |
| **Treatment regimens** | n=15 | n=204 |  |
| Chemotherapy-based | 1 (7%) | 17 (8%) |  |
| Hypomethylating Agent (HMA)-based | 10 (67%) | 166 (81%) | 0.18 |
| Chemo + HMA-based | 2 (13%) | 6 (3%) |  |
| HMA alone | 6 (40%) | 105 (52%) |  |
| HMA + other | 2 (13%) | 55 (27%) |  |
| Others | 0 | 3 (2%) |  |
| None | 4 (26%) | 18 (9%) |  |
| **Complete Blood Counts** |  |  |  |
| Absolute Neutrophil Count | 1.0 (0.3-9.5) | 1.1 (0-19.8) | 0.89 |
| Platelet count | 42 (7-241) | 49 (2-481) | 0.86 |
| Hemoglobin | 9 (8-12) | 9 (6-16) | 0.64 |
| Peripheral blood blast% | 4 (0-17) | 1 (0-25) | 0.31 |
| Bone marrow blast% | 6 (1-25) | 7 (0-28) | 0.51 |
| **Outcomes** |  |  |  |
| Dead | 7/17 (41%) | 185/253 (73%) | **0.010** |
| Transplant | 2/16 (12%) | 34/252 (13%) | 1 |
| Overall Response Rate | 7/9 (78%) | 98/171 (57%) | 0.31 |
| Complete Remission Rate | 2/9 (22%) | 51/171 (30%) | 1 |

VAF, Variant Allele Frequency; IPSS, R-IPSS, revised International Prognostic Scoring System
