# Supplementary figures and images for: Evolutionary action score identifies a subset of TP53 mutated myelodysplastic syndrome with favorable prognosis
Source: Blood Cancer J. 2021 Mar 6;11(3):52. doi: 10.1038/s41408-021-00446-y (PMC7936977; doi:10.1038/s41408-021-00446-y)

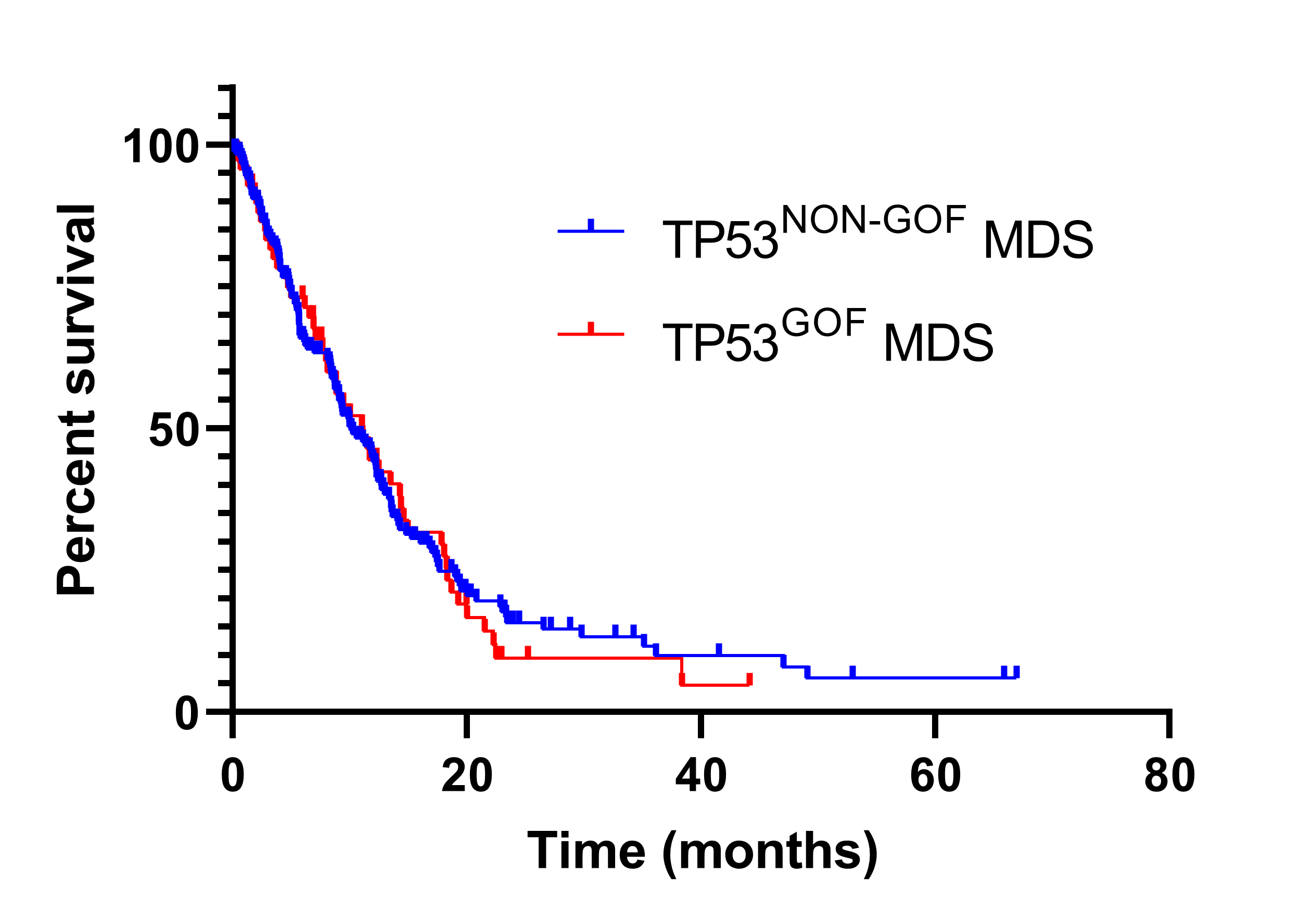

Supplement: Supplementary file 2 — Supplemental Figure S1. [file 41408_2021_446_MOESM2_ESM.tif]

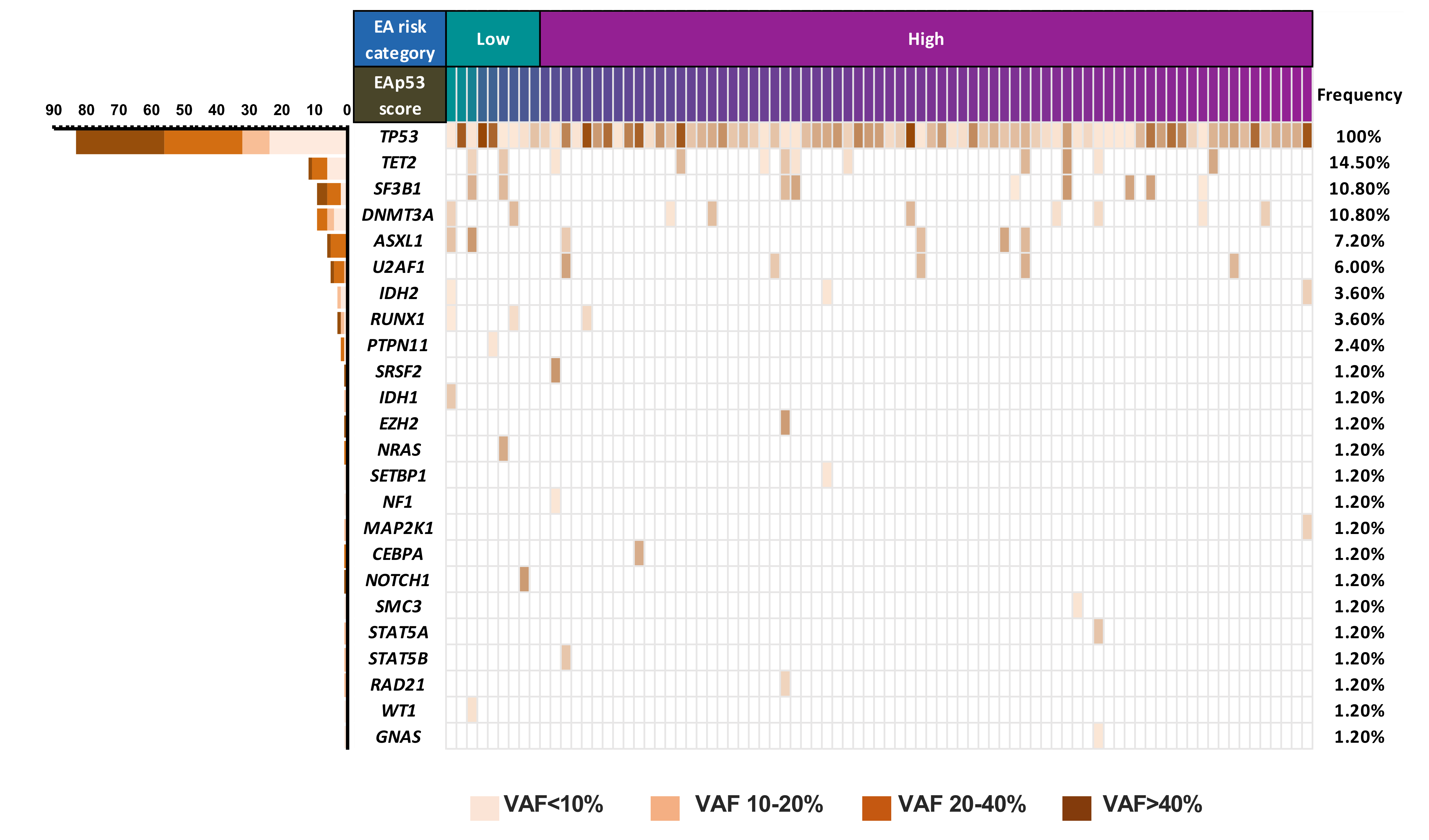

Supplement: Supplementary file 3 — Supplemental Figure S2. [file 41408_2021_446_MOESM3_ESM.tif]

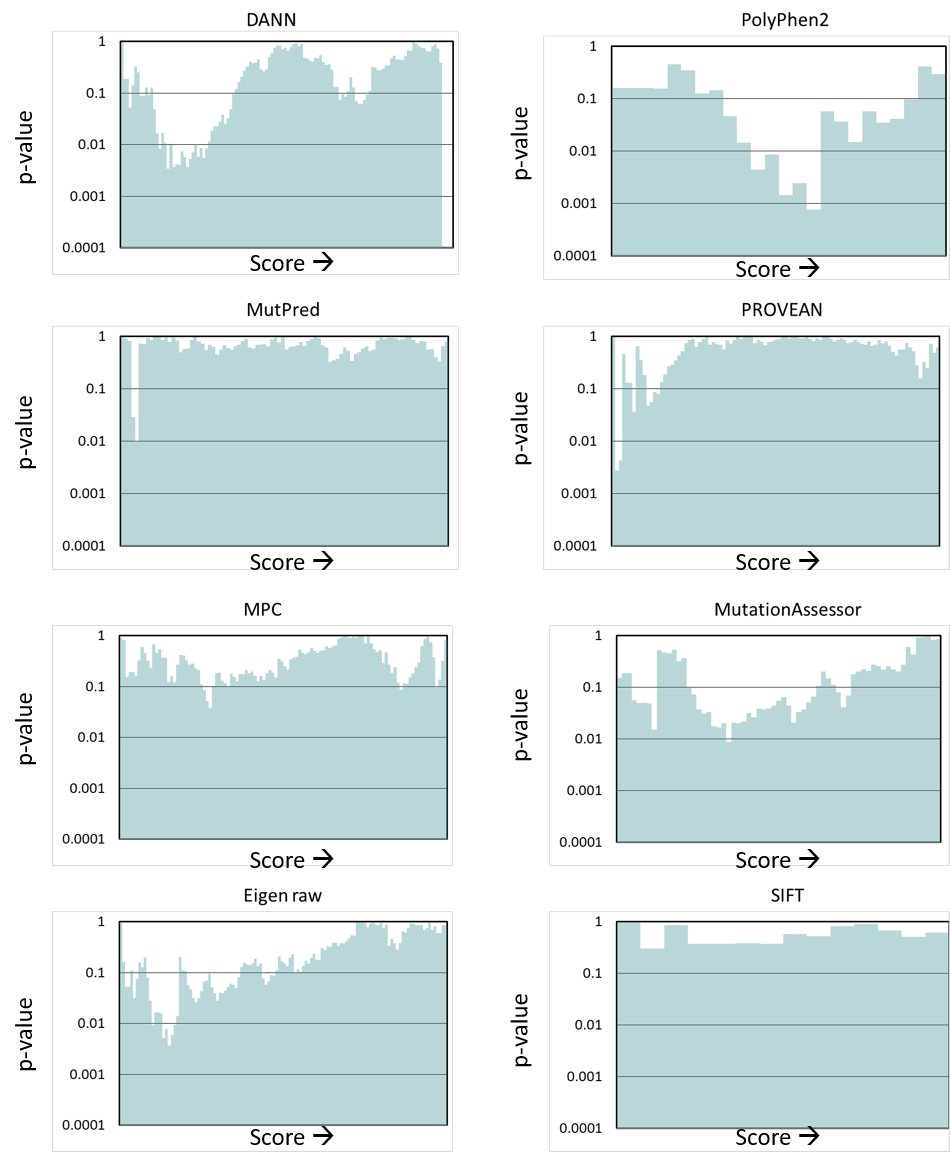

Supplement: Supplementary file 4 — Supplemental Figure S3. [file 41408_2021_446_MOESM4_ESM.tif]

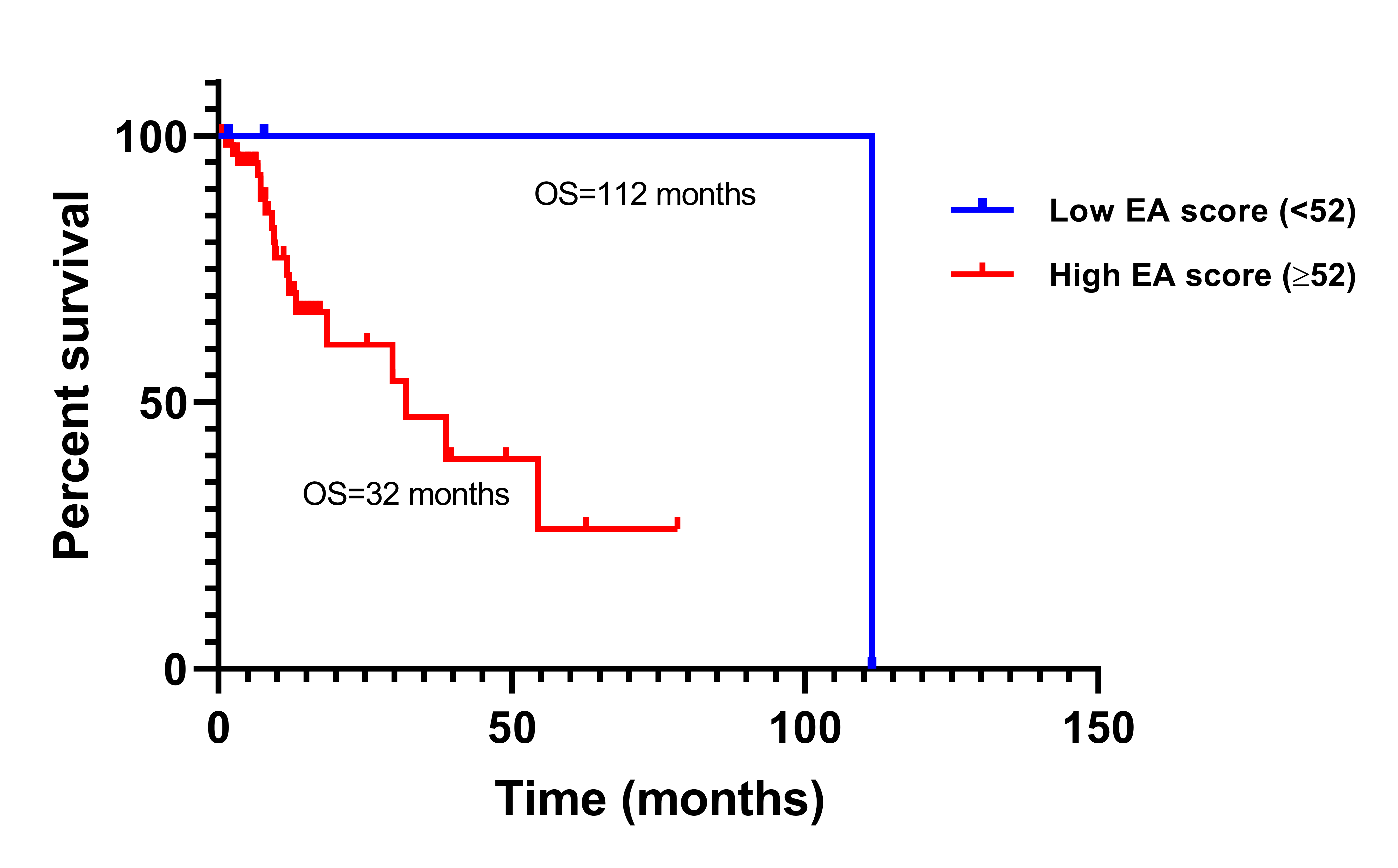

Supplement: Supplementary file 5 — Supplemental Figure S4. [file 41408_2021_446_MOESM5_ESM.tif]

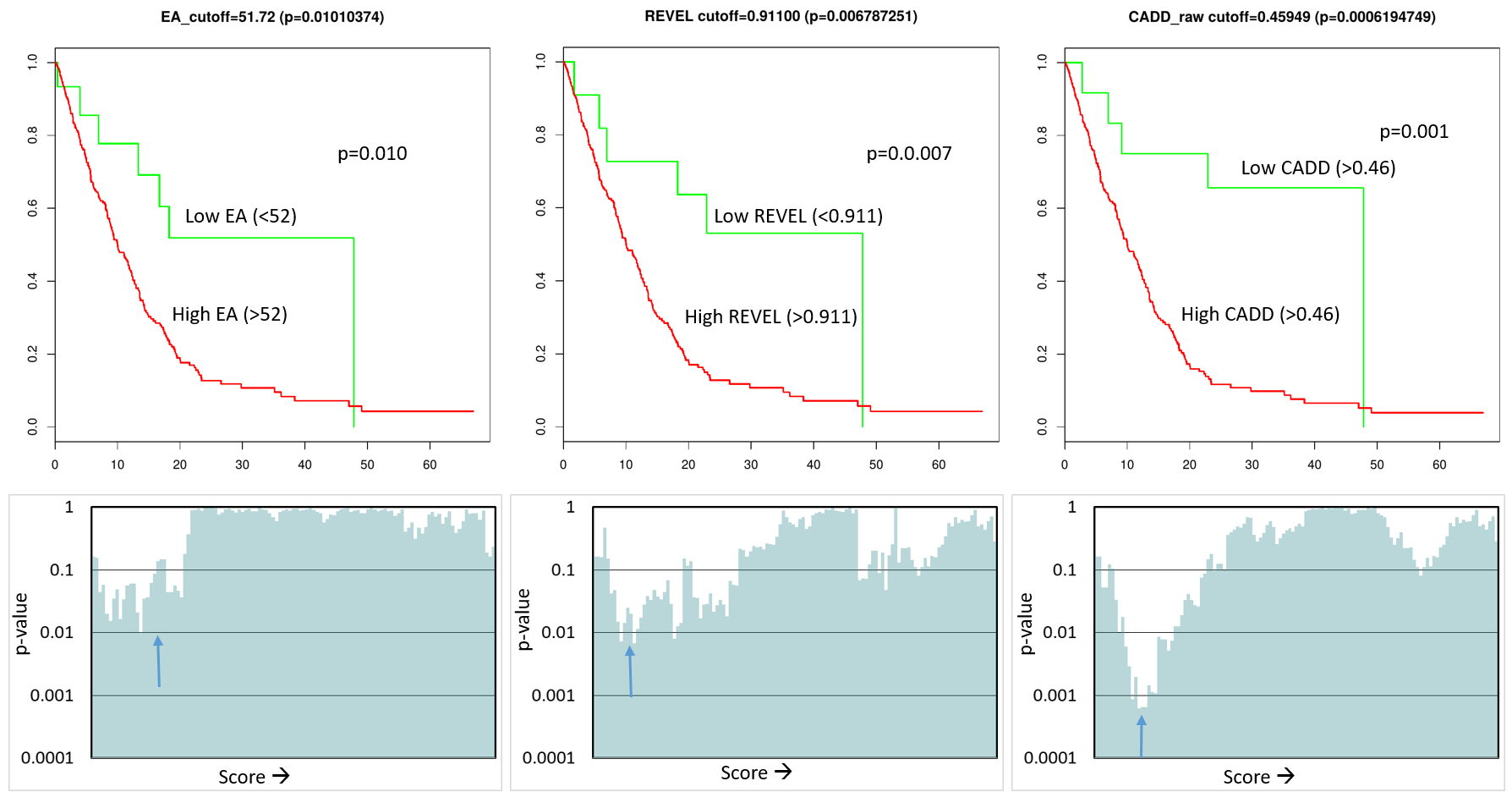

Supplement: Supplementary file 6 — Supplemental Figure S5. [file 41408_2021_446_MOESM6_ESM.tif]
